# Impact of Flavour Variability on Electronic Cigarette Use Experience: An Internet Survey

**DOI:** 10.3390/ijerph10127272

**Published:** 2013-12-17

**Authors:** Konstantinos E. Farsalinos, Giorgio Romagna, Dimitris Tsiapras, Stamatis Kyrzopoulos, Alketa Spyrou, Vassilis Voudris

**Affiliations:** 1 Onassis Cardiac Surgery Center, Sygrou 356, Kallithea 17674, Greece; E-Mails: dtsiapras@hotmail.com (D.T.); stkyrz@gmail.com (S.K.); aspirou@gmail.com (A.S.); vvoudris@otenet.gr (V.V.); 2 ABICH S.r.l, Biological and Chemical Toxicology Research Laboratory, Via 42 Martiri, 213/B-28924 Verbania (VB), Italy; E-Mail: giorgio.romagna@gmail.com

**Keywords:** electronic cigarette, flavours, smoking, tobacco, nicotine, smoking cessation, public health

## Abstract

*Background:* A major characteristic of the electronic cigarette (EC) market is the availability of a large number of different flavours. This has been criticised by the public health authorities, some of whom believe that diverse flavours will attract young users and that ECs are a gateway to smoking. At the same time, several reports in the news media mention that the main purpose of flavour marketing is to attract youngsters. The importance of flavourings and their patterns of use by EC consumers have not been adequately evaluated, therefore, the purpose of this survey was to examine and understand the impact of flavourings in the EC experience of dedicated users. *Methods:* A questionnaire was prepared and uploaded in an online survey tool. EC users were asked to participate irrespective of their current smoking status. Participants were divided according to their smoking status at the time of participation in two subgroups: former smokers and current smokers. *Results:* In total, 4,618 participants were included in the analysis, with 4,515 reporting current smoking status. The vast majority (91.1%) were former smokers, while current smokers had reduced smoking consumption from 20 to 4 cigarettes per day. Both subgroups had a median smoking history of 22 years and had been using ECs for 12 months. On average they were using three different types of liquid flavours on a regular basis, with former smokers switching between flavours more frequently compared to current smokers; 69.2% of the former subgroup reported doing so on a daily basis or within the day. Fruit flavours were more popular at the time of participation, while tobacco flavours were more popular at initiation of EC use. On a scale from 1 (not at all important) to 5 (extremely important) participants answered that variability of flavours was “very important” (score = 4) in their effort to reduce or quit smoking. The majority reported that restricting variability will make ECs less enjoyable and more boring, while 48.5% mentioned that it would increase craving for cigarettes and 39.7% said that it would have been less likely for them to reduce or quit smoking. The number of flavours used was independently associated with smoking cessation. *Conclusions:* The results of this survey of dedicated users indicate that flavours are marketed in order to satisfy vapers’ demand. They appear to contribute to both perceived pleasure and the effort to reduce cigarette consumption or quit smoking. Due to the fact that adoption of ECs by youngsters is currently minimal, it seems that implementing regulatory restrictions to flavours could cause harm to current vapers while no public health benefits would be observed in youngsters. Therefore, flavours variability should be maintained; any potential future risk for youngsters being attracted to ECs can be sufficiently minimized by strictly prohibiting EC sales in this population group.

## 1. Introduction

Cigarette smoking is considered the single most preventable cause of disease, affecting several systems in the human body and causing premature death [1]. The World Health Organisation predicts more than 1 billion deaths within the 21st century related to tobacco cigarettes [2]. Although there is overwhelming evidence for the benefits of smoking cessation [3], it is a very difficult addiction to break. Currently available nicotine replacement therapy have low long-term success rate, which may be attributed solely to psychological support [4], while oral medications are more effective [5] but are hindered by reports of adverse neuropsychiatric effects [6]. In this context, the tobacco harm reduction strategy has been developed, with a goal of providing nicotine through alternative methods in order to reduce the amount of harmful substances obtained by the user [7].

Electronic cigarettes (ECs) have been marketed in recent years as alternative to smoking products. They consist mainly of a battery and an atomiser where liquid is stored and gets evaporated by energy supplied to an electrical resistance. The liquid contains mainly propylene glycol and glycerol, with the option to include nicotine. A major characteristic of the EC liquid market is the availability of a variety of flavourings. Besides tobacco-like flavours, the consumer can choose flavours consisting of fruits, sweets, drinks and beverages and many more. The availability of so many flavours has been criticized by authorities such as the Food and Drug Administration (FDA), stating that there is a potential to attract youngsters [8]. Such a concern was probably raised by the experience with tobacco products, with studies showing that flavoured cigarettes were more appealing to young users [9]. A recent survey of electronic cigarette users found that almost half of participants were using non-tobacco flavours [10]. However, no survey was specifically designed to detect the impact of flavourings on EC experience by users. Therefore, the purpose of this survey was to evaluate the patterns of flavourings use and determine their popularity in a sample of dedicated adult EC users.

## 2. Methods

A questionnaire was prepared by the research team in two languages (English and Greek) and was uploaded in an online survey tool (www.surveymonkey.com). A brief presentation of the survey was uploaded in the website of a non-profit EC advocates group (www.ecigarette-research.com) together with informed consents in English and Greek. If the participant agreed with the informed consent, he was redirected to the questionnaire in the respective language by pressing the “I agree” button. The survey was available online for 15 days. The protocol was approved by the ethics committee of our institution. 

EC users of any age, irrespective of current or previous smoking status, were asked to participate to the survey. The survey was communicated in internet social media and several EC users’ forums and advocate groups worldwide. The IP address of the participants was recorded in order to remove double entries. There was an option for participants to report their email address for participation in future projects; unwillingness to report the email address was not a criterion for exclusion from the survey. Information about age, gender, country of residence and education level was requested. Past and present smoking status was asked and, based on the latter, participants were divided into two groups for the analysis: former smokers who had completely quit smoking and smokers who were still smoking after initiation of EC use. The questionnaire included questions about the type of flavours used regularly by the participants, whether the variety of flavourings was important in reducing or completely substituting smoking and defining the reasons for using multiple flavours. To assess difficulty in finding flavours of their preference at EC use initiation, the following question was asked: “Was it difficult to find the flavourings of your preference at initiation of EC use?”. The answers were scored as: 1, “not at all difficult”; 2, “slightly difficult”; 3, “difficult”; 4, “very difficult”; and 5, “extremely difficult”. To examine the importance of flavours variability in reducing or quitting smoking, the following question was asked: “Was the variability of flavourings important in your effort to reduce or completely substitute smoking?”. The answer was scored as: 1, “not at all important”; 2, “slightly important”; 3, “important”; 4, “very important”; and 5, “extremely important”. 

## 3. Statistical Analysis

Participants were categorised into current smokers and former-smokers according to their reported status at the time of participation to the survey. Results are reported for the whole sample and for each of the subgroups. The sample size varied by variable because of missing data. In some questions, responders were allowed to choose more than one option; in these cases, each answer is presented separately and the sum of responses may exceed 100%. Kolmogorov-Smirnoff tests were performed to assess normality of distribution of variables. Continuous variables are reported as median (interquartile range [IQR]). Categorical variables are reported as number (percentage). Mann Whitney U test was used to compare continuous variables between current and former smokers, while cross tabulations with χ^2^ test were used for categorical variables. Finally, a stepwise binary logistic regression analysis was performed, with smoking status (former *vs.* current smoker) as the independent variable and age, gender, education level, smoking duration, number of flavourings used regularly, and EC consumption (ml liquid or number of prefilled cartomisers) as covariates. A two-tailed *P* value of <0.05 was considered statistically significant, and all analyses were performed with commercially available statistical software (SPSS v. 18, Chicago, IL, USA).

## 4. Results

### 4.1. Baseline Characteristics

After excluding double entries, 4,618 participants were included to the analysis, with 4,515 reporting current smoking status (current *vs.* former smokers). The baseline characteristics of the study group and subgroups are displayed in Table 1. More than 90% were former smokers. The mean age was 40 years, with male predominance. No difference between former and current smokers was observed in age, while more males were former smokers. The vast majority were from America and Europe, with a small proportion residing in Asia and Australia. More than half of participants were educated to the level of university/college. Smoking duration was similar between subgroups. Interestingly, former smokers reported higher daily cigarette consumption before initiation of EC use, although the difference was not statistically significant. Current smokers reported a substantial reduction in cigarette consumption, from 20 to 4 cigarettes per day. The median duration of EC use was 12 months, with higher consumption (ml liquid or number of cartridges) reported by former smokers. Higher nicotine concentration liquids were used by current smokers (*P* = 0.005). In total, 140 participants (3.0%) reported using non-nicotine liquids, 2.8% of former and 1% of current smokers (χ^2^ = 4.5, *P* = 0.033); 21 users of non-nicotine liquids did not mention their current smoking status. Finally, more current smokers were using first (cigarette-like) and second generation (eGo-type) devices while more former smokers were using third generation devices (also called “Mods”, variable voltage or wattage devices). 

### 4.2. Perceptions in Relation to Flavours

Responses to questions related to flavours are displayed in Table 2. At the time of participation, most commonly used flavours were fruits, followed by sweets and tobacco. Significant differences were observed between subgroups. Characteristically, more current smokers were using tobacco flavours compared to former smokers, while more of the latter were using fruit and sweet flavours. On a regular basis, participants reported using 3 (IQR: 2–4) different types of flavours. At initiation of EC use, most popular flavours were tobacco followed by fruit and sweet flavours. The median score for difficulty to find the flavours of their preference at EC initiation was 2 (IQR: 1–3), with no difference between subgroups. Most participants (68.3%) were switching between flavours on a daily basis or within the day, with former smokers switching more frequently. More than half of the study sample mentioned that they like the variety of flavours and that the taste gets blunt from long-term use of the same flavour. The average score for importance of flavours variability in reducing or quitting smoking was 4 (“very important”). Finally, the majority of participants stated that restricting variability of flavours would make the EC experience less enjoyable while almost half of them answered that it would increase craving for tobacco cigarettes and would make reducing or completely substituting smoking less likely.

**Table 1:** Baseline characteristics of the study population and subgroups.

Characteristic	Total	Former Smokers	Current Smokers	Statistic	*P*
Participants, n (%)	4,618	4,117 (91.2)	398 (8.8)		
English translation	4,386 (95.0)	3,915 (95.1)	369 (92.7)		
Greek translation	232 (5.0)	202 (4.9)	29 (7.3)		
Region of residence, n (%)					
America	2,220 (48.5)	2,007 (48.7)	157 (39.4)		
Asia	76 (1.7)	58 (1.4)	16 (4.0)		
Australia	80 (1.7)	75 (1.8)	4 (1.0)		
Europe	2,197 (48.0)	1,939 (47.1)	217 (54.5)		
Education, n (%)					
High school or less	1,037 (22.7)	917 (22.3)	98 (24.6)		
Technical Education	1,099 (24.1)	993 (24.1)	86 (21.6)		
University/College	2,425 (53.2)	2,170 (52.7)	206 (51.8)		
Age (years)	40 (32–49)	40 (32–49)	40 (32–49)	U = 754,278	0.624
Gender (male)	3,229 (71.8)	2,922 (72.7)	246 (62.5)	χ^2^ = 18.0	<0.001
Smoking duration (years)	22 (15–30)	22 (15–30)	22 (14–30)	U = 816,534	0.924
Cigarette consumption before EC use (/d)	24 (20–30)	25 (20–30)	20 (19–30)	U = 768,398	0.189
Cigarettes consumption after EC use (/d)			4 (2–6)		
EC use duration (months)	12 (6–23)	12 (6–23)	12 (5–23)	U = 790,219	0.373
EC consumption (ml or cartridges/d)	4 (3–5)	4 (3–5)	3 (2–5)	U = 677,862	<0.001
Nicotine levels in EC (mg/ml)	12 (6–18)	12 (6–18)	12 (8–18)	U = 722,563	0.005
EC devices used, n (%)					
Cigarette-like	84 (1.8)	61 (1.5)	20 (5.0)	χ^2^ = 25.9	<0.001
eGo-type	1,123 (24.7)	966 (23.5)	133 (33.4)	χ^2^ = 19.5	<0.001
“Mods” ^a^	3,348 (73.5)	3,047 (74.0)	237 (59.5)	χ^2^ = 38.3	<0.001

Notes: Values presented as median (interquartile range) or number (percentage). Abbreviations: EC, electronic cigarette. ^a^ New generation devices, usually hand-made or with the ability to manually set the voltage or wattage delivery.

**Table 2:** Patterns of flavourings use in the study population and subgroups.

Characteristic	Total	Former Smokers	Current Smokers	Statistic	*P*
**Flavours used now, n (%) ^a^**					
Tobacco	1,984 (43.9)	1,773 (43.1)	211 (53.0)	χ^2^ = 14.6	<0.001
Mint/menthol	1,468 (31.8)	1,339 (32.5)	129 (32.4)	χ^2^ = 0.0	0.964
Sweet	2,836 (61.4)	2,629 (63.9)	207 (52.0)	χ^2^ = 21.8	<0.001
Nuts	691 (15.0)	643 (15.6)	48 (12.1)	χ^2^ = 3.5	0.060
Fruits	3,203 (69.4)	2,953 (71.7)	250 (62.8)	χ^2^ = 14.0	<0.001
Drinks/beverages	1,699 (36.8)	1,562 (37.9)	137 (34.4)	χ^2^ = 1.9	0.167
Other	1,028 (22.3)	946 (23.0)	82 (20.6)	χ^2^ = 1.2	0.281
**Flavours used at EC initiation, n (%) ^a^**					
Tobacco	3,118 (69.1)	2,846 (69.1)	272 (68.3)	χ^2^ = 0.1	0.746
Mint/menthol	1,086 (24.1)	1,004 (24.4)	82 (20.6)	χ^2^ = 2.8	0.092
Sweet	1,347 (29.8)	1,251 (30.4)	96 (24.1)	χ^2^ = 6.8	0.009
Nuts	203 (4.5)	186 (4.5)	17 (4.3)	χ^2^ = 0.1	0.821
Fruits	1,743 (38.6)	1,606 (39.0)	137 (34.4)	χ^2^ = 3.2	0.073
Drinks/beverages	808 (17.9)	748 (16.8)	60 (15.1)	χ^2^ = 2.4	0.124
Other	302 (6.7)	282 (6.8)	20 (5.0)	χ^2^ = 1.9	0.164
**Switching between flavours, n (%)**					
Daily/within the day	3,083 (68.3)	2,851 (69.2)	232 (58.3)	χ^2^ = 20.1	<0.001
Weekly	718 (15.9)	636 (15.4)	82 (20.6)	χ^2^ = 7.2	0.007
Less than weekly	465 (10.3)	412 (10.0)	53 (13.3)	χ^2^ = 4.3	0.038
At EC initiation, was it difficult to find the flavours of your preference? ^b^	2 (1–3)	2 (1–3)	2 (1–3)	U = 760,068	0.054
**Why do you feel the need to choose different flavours? n (%) ^a^**					
Like variety of choices	3,300 (73.1)	3,041 (73.9)	259 (65.1)	χ^2^ = 14.3	<0.001
They get “blunt” from long-term use	2,325 (51.5)	2,131 (51.8)	194 (48.7)	χ^2^ = 1.3	0.250
Other reasons	342 (7.6)	318 (7.7)	24 (6)	χ^2^ = 1.5	0.223
Was flavours variability important in reducing/quitting smoking? ^b^	4 (3–5)	4 (3–5)	4 (3–5)	U = 731,547	0.455
**How would your experience with EC change if flavours variability was limited? n (%) ^a^**					
Less enjoyable	3,111 (68.9)	2,886 (70.1)	225 (56.5)	χ^2^ = 31.2	<0.001
More boring	2,063 (45.7)	1,901 (46.2)	236 (40.7)	χ^2^ = 4.4	0.036
Increase craving for cigarettes	2,188 (48.5)	1,982 (48.1)	206 (51.8)	χ^2^ = 1.9	0.168
Less likely to reduce or quit smoking	1,793 (39.7)	1,617 (39.3)	176 (44.2)	χ^2^ = 3.7	0.054
No difference	285 (6.3)	253 (6.1)	32 (8.0)	χ^2^ = 2.2	0.138

Notes: Values presented as median (interquartile range) or number (percentage). Abbreviations: EC, electronic cigarette. ^a ^Participants were allowed to choose more than one answers. ^b^ Score reported (see text for details).

Binary logistic regression analysis showed that male gender (B = 0.373, *P* = 0.001), EC consumption (B = 0.046, *P* = 0.044) and number of flavours regularly used (B = 0.089, *P* = 0.038) were associated with complete smoking abstinence in this population of dedicated long-term vapers, while age, education level and smoking duration were not associated with smoking abstinence. 

## 5. Discussion

This is the first survey that specifically focused on the issue of flavours and their impact in EC use. A substantial number of dedicated EC consumers participated; they reported that flavours play an important role in their EC use experience and in reducing cigarette consumption and craving, while the number of flavours regularly used was independently associated with complete smoking abstinence in this population.

The availability of a variety of flavours has been a controversial issue since the initial appearance of ECs to the market. Most companies offer a variety of flavours, from those resembling tobacco to a large number commonly used in the food industry. Public health authorities have raised concerns about this issue, and several statements have been released suggesting flavours could attract youngsters [8,11,12]. Such concerns are probably rooted back to the marketing of the tobacco industry for flavoured tobacco cigarettes. Internal industry documents and published surveys indicated that flavoured tobacco products are more appealing to youngsters and may be a gateway to maintaining smoking as a long term habit, while use by adults was quite low [13,14,15,16]. This is the main reason why the FDA decided to implement a ban on characteristic flavours in tobacco cigarettes [17]. It was expected that such concerns would be raised for ECs, although current vapers are overwhelmingly adults. Anecdotal evidence from EC consumers’ internet forums and results from surveys [10] have shown that different flavours are very popular among dedicated users. The results of this survey confirm previous observations by finding that dedicated users switch between flavours frequently and the variability of flavours plays an important role both in reducing cigarette craving and in perceived pleasure. Moreover, the number of flavours used was associated with smoking cessation. Therefore, flavours variability is needed to support the demand by current vapers, who are in their vast majority adults. This survey also indicated that there is a switch in flavours preference of EC consumers; tobacco is the preferred flavour when initiating EC use, probably because smokers are used to this flavour and feel the need to use something that resembles their experience from smoking. However, different choices are made as time of use progresses. This may be a way to distract them from the tobacco flavour in order to reduce smoking craving; alternatively, it could indicate that they just don’t need the tobacco flavour any more, but feel the desire to experiment with new flavours. In some cases, tobacco flavour may even become unpleasant, especially in those who have completely quit smoking. The improvement in olfactory and gustatory senses in these people can lead to both more pleasure perceived from different flavours and an aversion to tobacco flavour (in a similar way that it is unpleasant for a non-smoker); the latter has been reported in EC consumers’ forums (http://www.e-cigarette-forum.com/forum/polls/209041-do-you-vape-tobacco-flavors.html). Such a phenomenon may contribute to lower relapse to smoking and may prevent the EC from being a gateway to smoking; however, this should be specifically studied before making any conclusions. Finally, the issue of taste buds “tolerance”, which is anecdotally mentioned by vapers, was reported by almost half of the sample as a reason to switch between flavours, although it is most probably a type of olfactory rather than gustatory tolerance.

Besides information on the use of flavourings, this survey provides information on other issues related to EC use. A small minority of participants were using first generation cigarette-like devices. This has been observed in other surveys [10]. There was a higher prevalence of third-generation devices used in the subgroup of former smokers compared to current smokers. Such devices have the ability to provide higher energy to the atomiser, thus producing more vapour and delivering more pleasure to the user [18,19]. Until now, two randomised studies evaluating the efficacy of EC use in smoking cessation have used first-generation cigarette-like devices [20,21]. It is possible that newer generation devices may be more effective in substituting smoking, and this should be evaluated in future studies. Additionally, former smokers were using lower nicotine-concentration liquids compared to current smokers. It has been observed from previous studies that EC users who have completely substituted smoking try to gradually reduce their nicotine use [18]. Despite that, only 2.8% of former smokers were using 0-nicotine liquids at the time of survey participation, indicating that nicotine is important in smoking abstinence and that EC consumers remain long-term nicotine users. However, the possibility that several vapers may quit EC use shortly after switching to non-nicotine liquids cannot be excluded; such users would not participate to this survey, therefore overestimating the significance of nicotine on EC use. Finally, we observed a male predominance in participation to this survey, which is in line with previous studies [10,18]. In this survey, males were more likely to have completely quit smoking. Further studies are needed to explore this phenomenon and define whether females are less successful in smoking cessation with EC use, are less motivated long-term users or use ECs in the short term as smoking substitutes.

There are some limitations applicable to this study. The survey was announced and promoted in popular EC websites. Therefore, it is expected that dedicated users with positive experience with ECs would mainly participate, and the high proportion of former smokers confirms this. However, it is important to evaluate the patterns of use in smokers who have successfully quit smoking, since this can provide health officials with information on how to educate smokers into using ECs, especially during the initial period of use. Although a significant proportion stated that flavours play a major role in reducing or quitting smoking, this study was not designed to evaluate whether variability of flavours may promote smoking cessation in the general population; moreover our sample is not representative of the general population of smokers, who are generally less educated compared to the population evaluated here [22]. This should be evaluated in a randomised study. Finally, although the fact that flavours are important for existing EC users provides sufficient explanation for their current marketing, it does not exclude the possibility that they may also attract youngsters. However, currently available evidence indicates that regular use of ECs by non-smoking adults or youngsters is very limited [23,24,25]; thus, any restriction of flavours for the reason of protecting youngsters is currently not substantiated by evidence and no public health benefit would be derived. On the contrary, such a measure could have a negative impact and cause harm in current vapers, who are reporting that they enjoy flavours and that restrictions would make smoking reduction or cessation more difficult and would increase cigarette craving. Therefore, it would be more realistic and valuable to promote restrictions to the use of ECs by youngsters and to properly inform the public that ECs should be used only by smokers as a method to reduce cigarette consumption or completely substitute smoking.

## 6. Conclusions

The results of this survey indicate that EC liquid flavourings play a major role in the overall experience of dedicated users and support the hypothesis that they are important contributors in reducing or eliminating smoking consumption. This should be considered by the health authorities; based on the current minimal adoption of ECs by youngsters, it is reasonable to support that any proposed regulation should ensure that flavourings are available to EC consumers while at the same time restrictions to the use by youngsters (especially non-smokers) should be imposed in order to avoid future penetration of EC use to this population.
